# Impact of Diabetes Mellitus on Lower Urinary Tract Symptoms in Benign Prostatic Hyperplasia Patients: A Meta-Analysis

**DOI:** 10.3389/fendo.2021.741748

**Published:** 2022-02-01

**Authors:** Caihong Xin, Huaying Fan, Jing Xie, Jingcheng Hu, Xin Sun, Qiuchen Liu

**Affiliations:** ^1^ Department of Endocrinology and Metabolism, Fourth People’s Hospital of Shenyang, Shenyang, China; ^2^ Department of Endocrinology and Metabolism, First Affiliated Hospital of Soochow University, Suzhou, China; ^3^ Department of Urology, First Affiliated Hospital of Soochow University, Suzhou, China

**Keywords:** diabetes mellitus, LUTS, benign prostatic hyperplasia, meta-analysis, lower urinary tract symptoms

## Abstract

**Background:**

Benign prostatic hyperplasia (BPH) is a disease that causes lower urinary tract symptoms (LUTS), which are the most common urological problem in approximately one-third of the male population aged over 50 years. Some studies have suggested that diabetes may be a risk factor for the development of BPH. However, whether diabetes aggravates the LUTS of BPH patients is still controversial.

**Aim:**

To investigate the impact of diabetes mellitus on LUTS in BPH patients.

**Methods:**

A literature search was conducted using Web of Science, Embase, PubMed, and China National Knowledge Infrastructure literature databases. This meta-analysis was registered in PROSPERO (registration number: CRD 42020200794). Fixed- or random-effects models were used for analysis according to heterogeneity. The results of the systematic analysis are presented as weighted mean difference (WMD) with the corresponding 95% confidence intervals (CI).

**Results:**

In total, 1308 studies were retrieved from databases and 18 articles comprising 1685 cases and 4653 controls were selected for meta-analysis. The results of the meta-analysis showed that the International Prostate Symptom Score (IPSS) value and prostate volume of BPH patients with diabetes was significantly higher than that of BPH patients without diabetes.

**Conclusions:**

This systematic review is the first to evaluate the impact of diabetes mellitus on LUTS in BPH patients. The results of our meta-analysis support the hypothesis that LUTS in BPH patients is increased in patients with diabetes mellitus compared with controls, which suggests that physicians should pay more attention to BPH patients with diabetes mellitus.

**Systematic Review Registration:**

PROSPERO [https://www.crd.york.ac.uk/PROSPERO/display_record.php?RecordID=200794], identifier CRD 42020200794.

## Introduction

Benign prostatic hyperplasia (BPH) is a disease that causes lower urinary tract symptoms (LUTS), which are the most common urological problem in approximately one-third of the male population aged over 50 years (1). Age, sex hormones, diet, diabetes, obesity, and genetic factors are closely related to the occurrence of BPH (2). Typically, clinicians treat BPH and type 2 diabetes as separate entities, although some have suggested that diabetes may be a risk factor for the development and progression of BPH (3, 4). Vascular damage and atherosclerosis caused by diabetes mellitus can aggravate the ischemia of the prostate, and insulin-like growth factor can increase the risk of prostate hyperplasia and prostate cancer (5).

However, under clinical observation, whether diabetes aggravates the LUTS of BPH patients is still controversial. Bang et al. (6) showed that the LUTS of BPH patients with diabetes mellitus are more obvious than those of BPH patients alone, while Boon et al. (7) showed that the International Prostate Symptom Score (IPSS) value of BPH patients without diabetes mellitus is higher than that of BPH patients with diabetes mellitus.

Therefore, the aim of this systematic review and meta-analysis is to investigate the impact of diabetes mellitus on LUTS in BPH patients.

## Methods

### Search

We searched the following electronic databases: Web of Science, Embase, PubMed, and China National Knowledge Infrastructure. The following search terms were identified in the title or abstract: (diabetes mellitus[Title/Abstract]) AND (((((lower urinary tract symptoms [Title/Abstract]) OR benign prostatic enlargement[Title/Abstract]) OR benign prostatic hyperplasia[Title/Abstract]) OR prostate[Title/Abstract]) OR LUTS[Title/Abstract]). All studies published from 1980 to 2020 were included in the search. The references of the retrieved articles were checked to determine other eligible studies. Unpublished studies were not included. The search languages were limited to English and Chinese. This systematic review and meta-analysis was registered in PROSPERO (registration number: CRD 42020200794). A complete list of preferred reporting items for system reviews and meta-analysis is provided in the supplementary data (**Table S1**).

### Inclusion Criteria

Inclusion of the selected studies was based on the following criteria: (1) a case-control design was used; (2) participants of the control group were BPH patients without diabetes mellitus and participants of the case group were BPH patients with diabetes mellitus; (3) sufficient data for cases and controls were provided to enable calculation of the weighted mean difference (WMD) with the corresponding 95% confidence intervals (CI) and *P* values.

### Outcome Indicators

(1) IPSS score. IPSS score is one of the most important indicators used to judge the severity of LUTS in patients with BPH. It is mainly composed of 7 symptoms: incomplete emptying, frequency, intermittency, urgency, weak stream, straining and nocturia. The answers to this question range from “delighted” to “terrible” or 0 to 6, respectively (8). (2) Prostate volume (PV, ml); (3) Maximal flow rate (Qmax, ml/s); (4) Prostate-Specific Antigen (PSA, ng/ml). Other baseline characteristics were also recorded.

### Data Extraction and Risk of Bias

Two researchers independently extracted general information from the included articles such as the first author, year of publication, region, population, number of cases and controls, and outcomes in the cases and controls. The Newcastle-Ottawa Scale (NOS) is a risk of bias assessment tool for observational studies that is recommended by the Cochrane Collaboration (9). NOS ranged from zero to nine stars. Quality was based on star scores, with 7-9 stars indicating high quality, 4-6 stars middle quality and 0-3 stars low quality. The two researchers independently assessed the studies by discussion, compared their findings, and resolved any differences by consensus. If no consensus could be reached, a third researcher was commissioned to resolve the difference.

### Statistical Analysis

The results of the systematic analysis are presented as Weighted Mean Difference (WMD) and 95% CI. Heterogeneity among studies was assessed using Cochran’s Q test and the I^2^ statistic. If I^2^ was < 50%, it was considered to have a low or moderate heterogeneity, and a fixed-effect model (Inverse variance Method) was used. Otherwise, heterogeneity was considered high and a random-effect model (Inverse variance heterogeneity Method) was used for analysis. Subgroup analyses were also performed. Additionally, we performed a sensitivity analysis to evaluate the influence of any given study on the pooled estimate. Publication bias was evaluated using Begg’s and Egger’s tests. Significance level was determined by a *P* value of < 0.05. All statistical analyses were performed using the Stata version 12.0 (College Station, Texas, USA).

## Results

**Figure 1:**
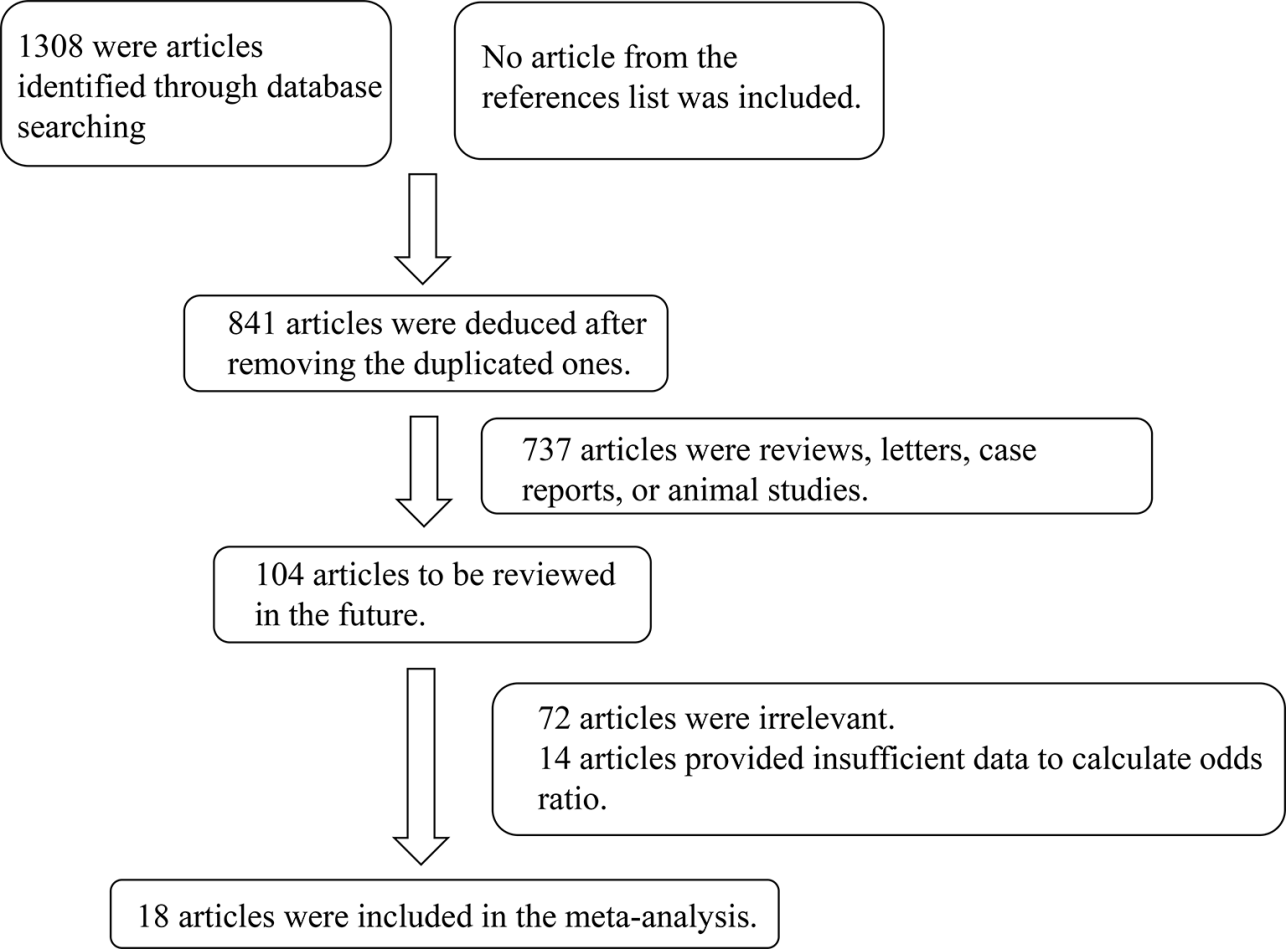
Flow chart showing the detailed procedure for the inclusion or exclusion of studies.

In total, 1308 studies were retrieved from the PubMed, Web of Science, Embase, and CNKI databases. No article from the references list was included. After screening, a total of 18 articles comprising 1685 cases and 4653 controls were selected for meta-analysis (6, 7, 10–25). The inclusion criteria during the full-text selection are shown in **Figure 1**. The characteristics of the selected studies are summarized in **Table 1**. The summary of risks was presented in **Table S2**.

**Table 1:** The characteristic of the selected studies in the meta-analysis.

Study	Publication Year	Study Period	Region	Case (n)	Control (n)	Age (years)	Outcome		NOS score
Michel M	2000	–	Germany	64	53	Case: 68.4 ± 0.02 Control: 64.7 ± 0.01	IPSS, PV, Qmax	6	
Boon T	2001	1993 - 2000	Netherlands	32	565	Case: 64 ± 9 Control: 66 ± 8	IPSS, PV, Qmax	7	
Berger A	2005	2003.9 - 2004.9	Australia	28	24	Case: 53 - 68 Control: 58 - 69	IPSS, Qmax, PSA	8	
Sarma A	2008	1990 and 1996	USA	170	2314	56.0 ± 10.5	PV, Qmax, PSA	6	
Liu N	2010	2004.12 - 2008.4	China	40	56	Case: 70.0 ± 10.3 Control: 73.0 ± 9.6	IPSS, Qmax	5	
Ding J	2010	2006.12 - 2008.6	China	47	59	63.21 ± 7.18	IPSS, Qmax	6	
Palida A	2011	2009.12 - 2010.8	China	90	100	Case: 70.38 ± 7.9 Control: 71.74 ± 9.2	IPSS, PV, Qmax, PSA	7	
Xie NZ	2013	2010.1 - 2012.10	China	103	64	80 - 92	IPSS, PV, PSA	6	
Qu XB	2014	2008.2 - 2009.3	China	64	53	77.74 ± 5.64	IPSS, PV, PSA	7	
Bang W	2014	2010.4 - 2012.6	Korea	139	139	65.33 ± 9.05	IPSS, PV, Qmax, PSA	9	
Yuan L	2015	2011.1 - 2013.12	China	42	48	68.57 ± 5.83	IPSS, Qmax	5	
Wang B	2017	2011.2 - 2012.5	China	400	330	59.32 ± 5.47	IPSS, PV, PSA	7	
Ozcan L	2017	2008 - 2012	Turkey	100	200	68.2 ± 7.4	IPSS, PV, Qmax, PSA	7	
Xu J	2017	2012.4 - 2015.6	China	63	52	76.8 ± 2.9	IPSS, PV, PSA	7	
Zhao Z	2018	2017.1 - 2018.1	China	40	40	Case: 65.4 ± 6.81 Control: 68.7 ± 8.97	IPSS, PV, Qmax, PSA	6	
Gao YS	2018	2015.4 - 2016.4	China	183	469	75.36 ± 5.55	IPSS, PV, PSA	7	
Liu YD	2018	2014.1 - 2017.7	China	30	37	70.2 ± 8.3	IPSS, Qmax	6	
Li YC	2019	2016.1 - 2018.10	China	50	50	Case: 67.8 ± 7.7 Control: 69.3 ± 2.1	IPSS, PV, Qmax, PSA	7	

IPSS, International Prostate Symptom Score; PV, Prostate Volume; Qmax, Maximal Flow Rate; PSA, Prostate-Specific Antigen; NOS, Newcastle-Ottawa Scale.

### Results of the Meta-Analysis

**Figure 2:**
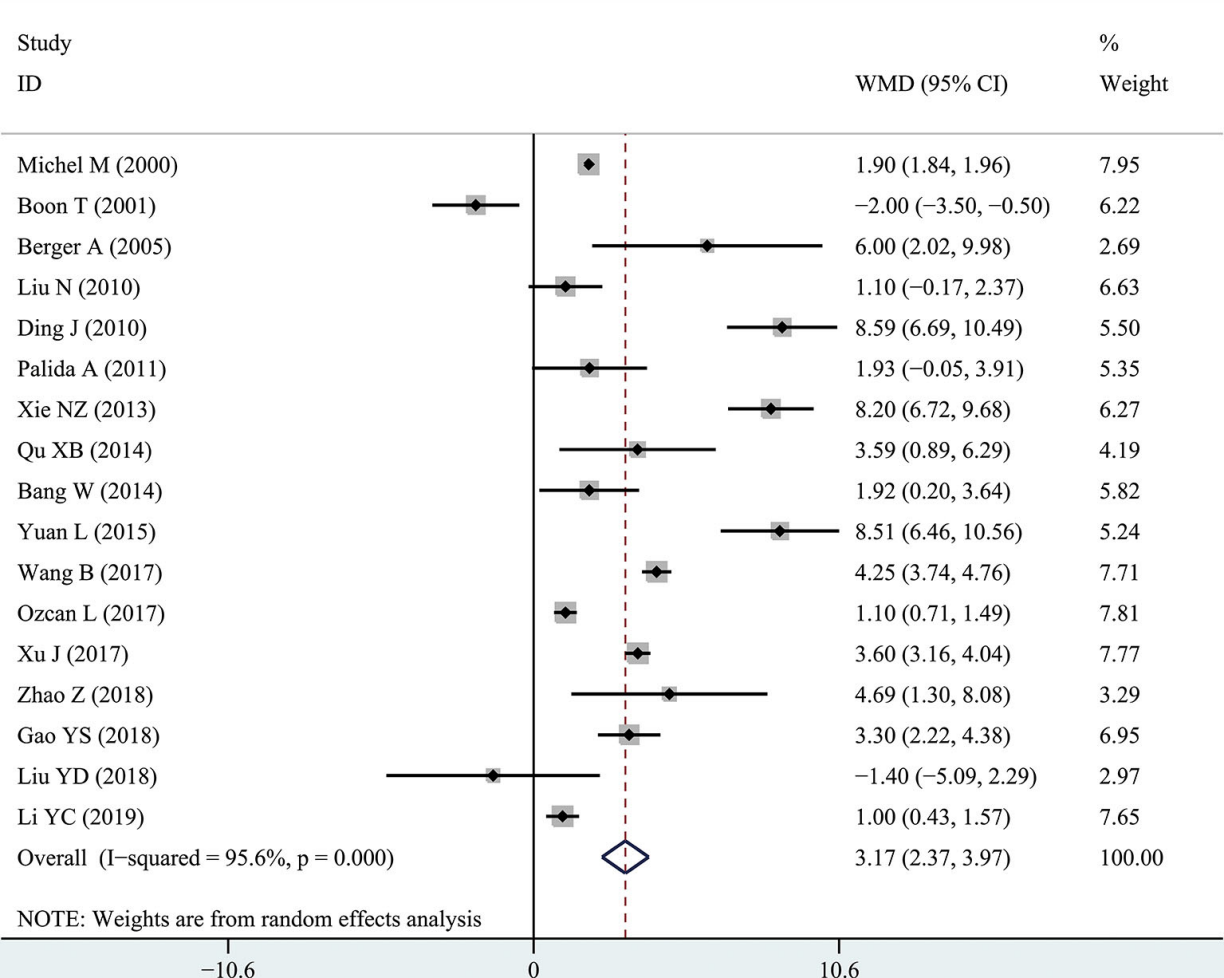
Forest plots of International Prostate Symptom Score comparing diabetes group to without diabetes group in benign prostatic hyperplasia patients. The diamond represents the pooled Weighted Mean Difference (WMD) and 95% confidence intervals (CI).

**Figure 3:**
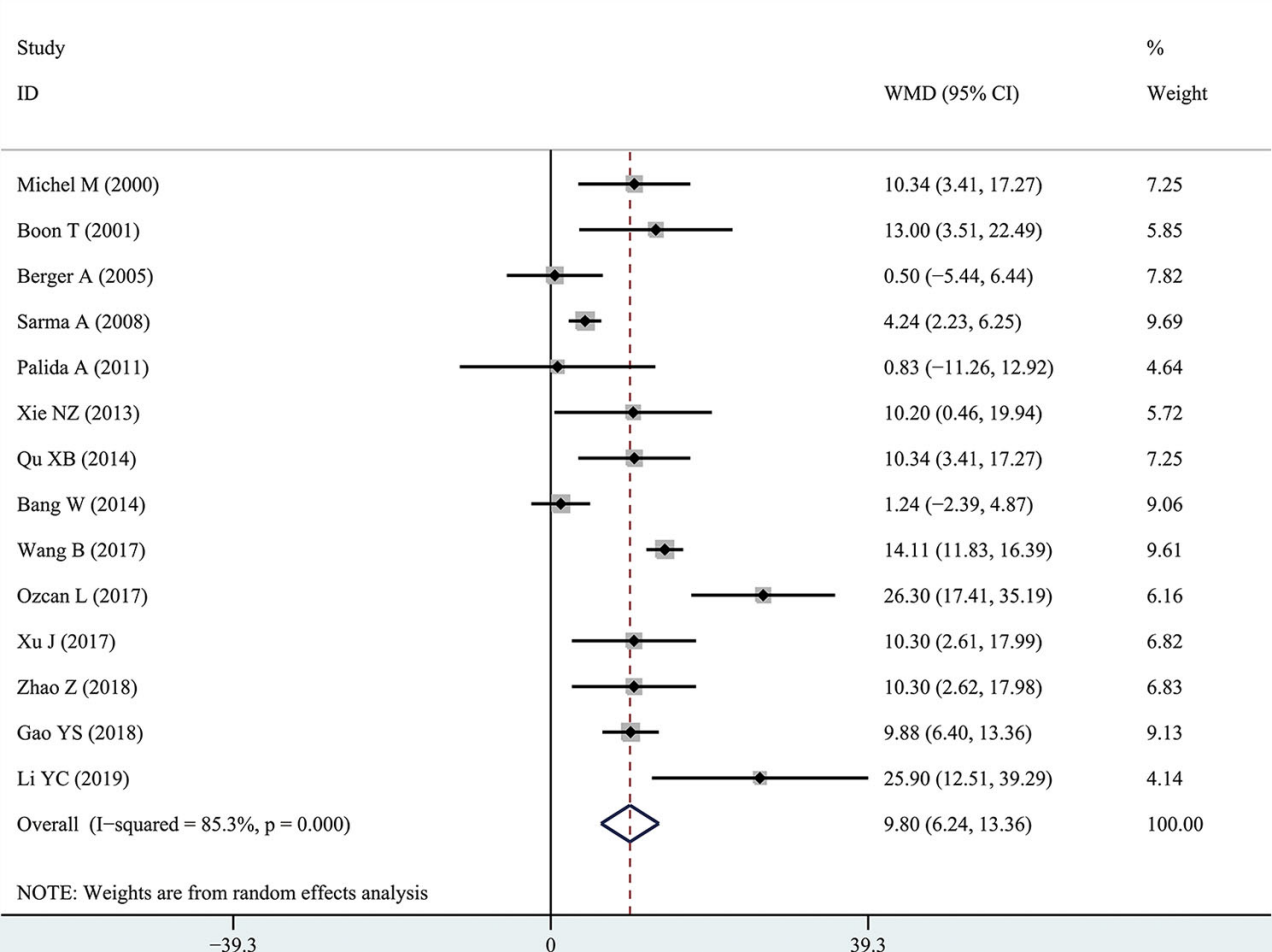
Forest plots of prostate volume comparing diabetes group to without diabetes group in benign prostatic hyperplasia patients. The diamond represents the pooled Weighted Mean Difference (WMD) and 95% confidence intervals (CI).

**Figure 4:**
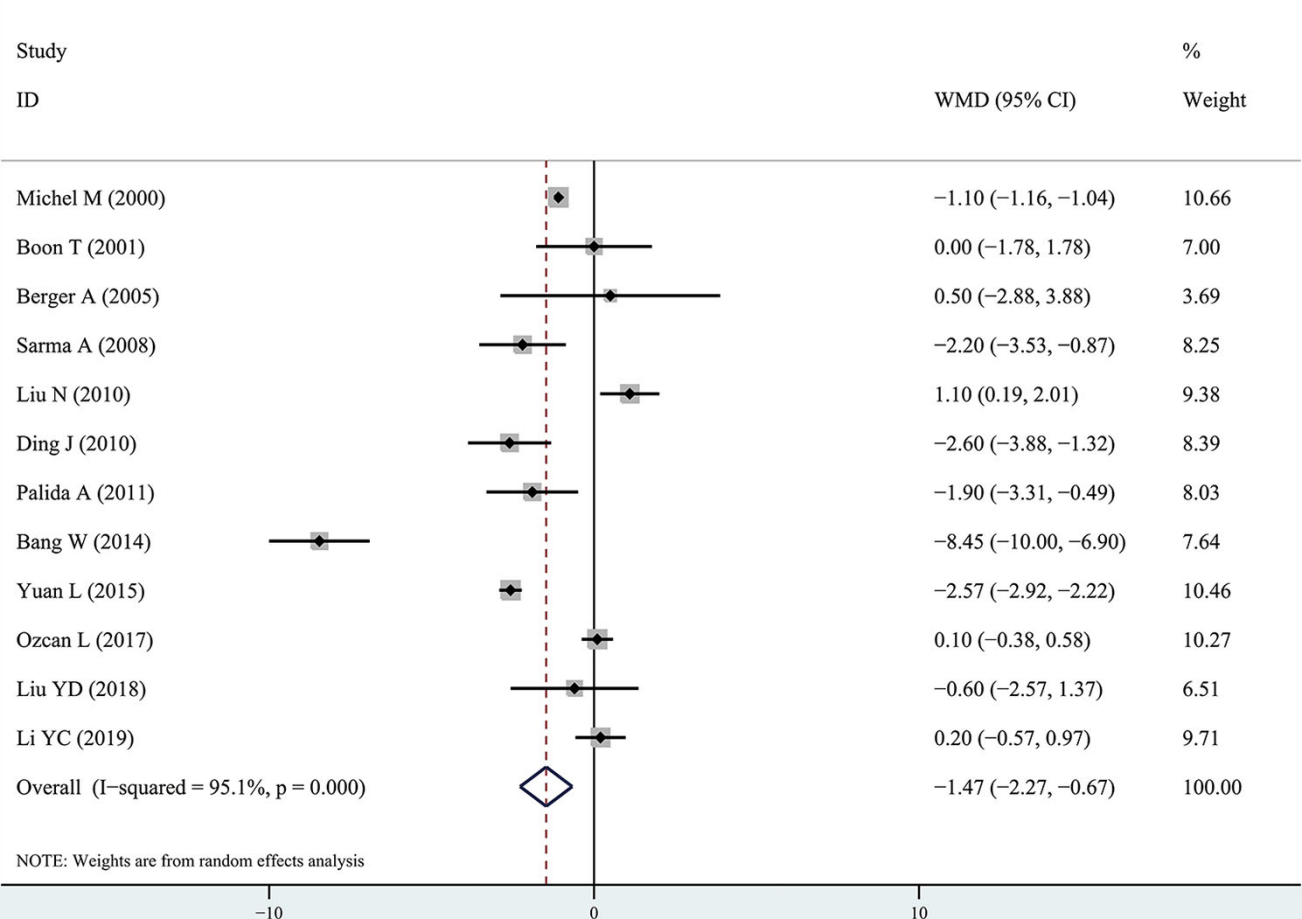
Forest plots of maximal flow rate comparing diabetes group to without diabetes group in benign prostatic hyperplasia patients. The diamond represents the pooled Weighted Mean Difference (WMD) and 95% confidence intervals (CI).

**Figure 5:**
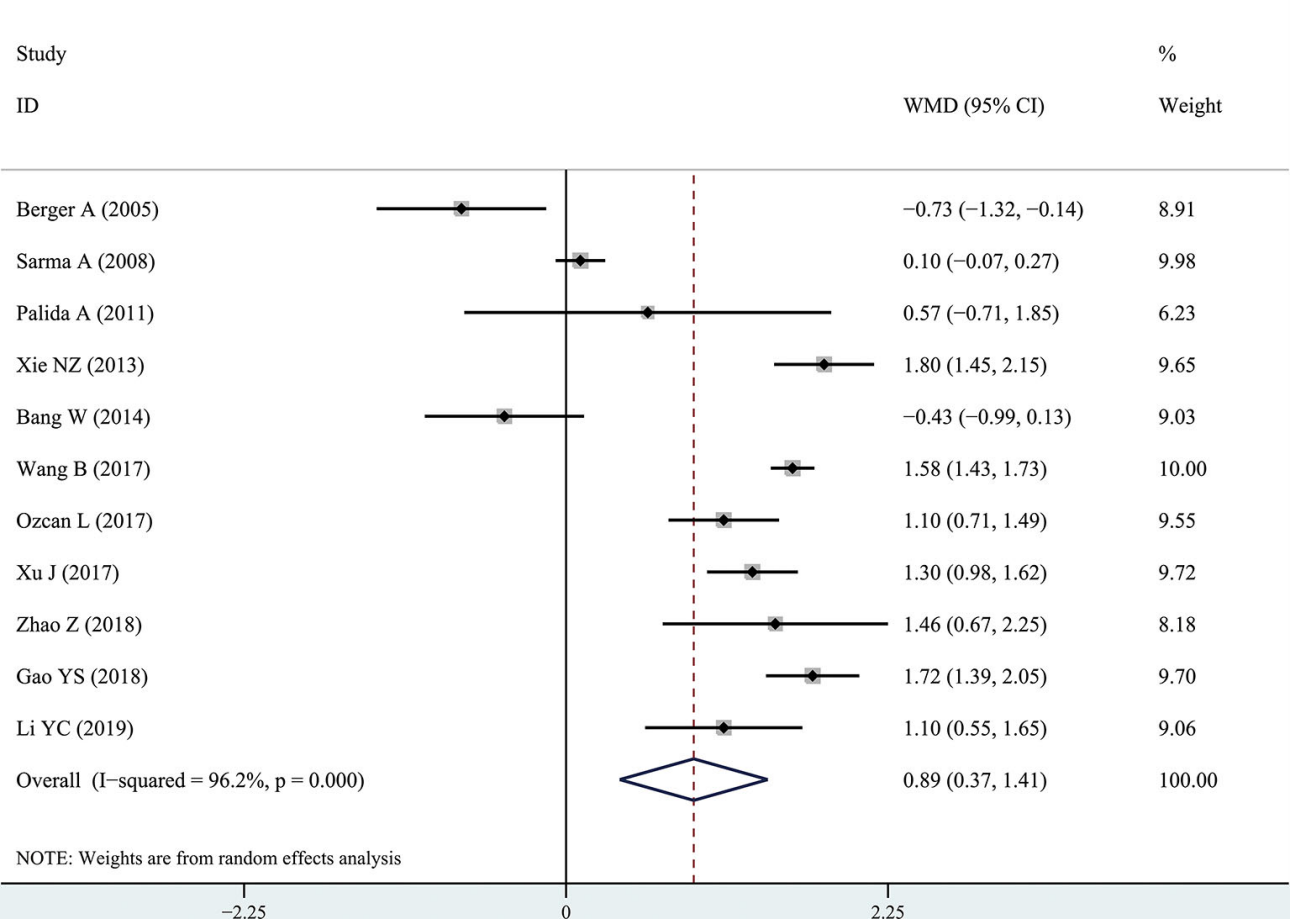
Forest plots of prostate-specific antigen value comparing diabetes group to without diabetes group in benign prostatic hyperplasia patients. The diamond represents the pooled Weighted Mean Difference (WMD) and 95% confidence intervals (CI).

The results of the meta-analysis showed that the IPSS value of BPH patients with diabetes was significantly higher than that of BPH patients without diabetes (WMD: 3.17, 95% CI [2.37, 3.97]). The forest plots of IPSS value in patients with diabetes compared with controls are presented in **Figure 2**. The prostate volume of BPH patients with diabetes was also significantly higher than that of BPH patients without diabetes (WMD: 9.80, 95% CI [6.24, 13.36]) (**Figure 3**). Furthermore, we investigated the Qmax of BPH patients between different groups. The Qmax of BPH patients with diabetes was significantly lower than that of BPH patients without diabetes (WMD: -1.47, 95% CI [-2.27, -0.67]) (**Figure 4**). The forest plots of PSA in patients with diabetes compared with the controls are presented in **Figure 5** (WMD: 0.89, 95% CI [0.37, 14.1]). We also performed the subgroup analysis based on different races, the results were presented in **Table 2**.

**Table 2:** The results of different outcomes according to the races.

	IPSS value		PV		Qmax		PSA	
	No. of studies	WMD, 95% CI	No. of studies	WMD, 95% CI	No. of studies	WMD, 95% CI	No. of studies	WMD, 95% CI
Asian	13	3.85[2.65, 5.05]	9	9.81[5.55, 14.07]	7	-2.08[-3.86, -0.30]	8	1.21[0.82, 1.61]
Caucasian	4	1.03[0.04, 2.10]	5	10.09[2.98, 17.20]	5	-0.71[-1.58, 0.17]	3	0.18[-0.64, 1.01]

IPSS, International Prostate Symptom Score; PV, Prostate Volume; Qmax, Maximal Flow Rate; PSA, Prostate-Specific Antigen; WMD, Weighted Mean Difference; CI, Confidence Intervals.

### Sensitivity Analysis and Publication Bias

Sensitivity analysis was performed to examine the influence of each study. We found no significant difference between the results of the sensitivity analysis and our previous estimates, indicating that our statistical results were relatively credible (**Figures S1** **–** **S4**). The articles obtained from the databases were carefully and comprehensively searched. Begg’s and Egger’s tests were also conducted to determine whether potential publication bias existed in the reviewed literature. The Begg’s test results of IPSS value, PV, Qmax, and PSA were 0.537, 0.622, 0.631, and 0.161, respectively, and Egger’s test results were 0.124, 0.433, 0.688, and 0.746, respectively. They suggested that there was no publication bias. The funnel plots of IPSS value, PV, Qmax, and PSA were presented in **Figures S5** **–** **S8**, respectively.

## Discussion

BPH is one of the most common urological problems in elderly men. Mauro et al. found that metabolic syndrome patients might have higher prostate volume, while there was no difference of IPSS score compared to the controls. However, in their meta-analysis, they only included 8 articles (26). This meta-analysis of 18 independent studies is the first to investigate the impact of diabetes mellitus on LUTS in BPH patients. Our results showed that the risk of LUTS was significantly higher in BPH patients with diabetes than in the BPH alone group.

IPSS score is one of the most important indicators used to judge the severity of LUTS in patients with BPH (27). The results of this analysis showed that the IPSS score of the diabetic BPH group was significantly higher than that of the non-diabetic BPH group, indicating that diabetes mellitus may aggravate the LUTS of BPH patients. This may be that hyperglycemia can cause an increase of free calcium ions in smooth muscle and neuronal cell solute and increase sympathetic nerve activity, thus enhancing the contractile activity of prostate smooth muscle (28). Diabetes can inactivate nerve growth factor (NGF) transported by the axon of the afferent bladder detrusor pathway, and hyperglycemia can cause excess oxygen-free radicals which damage the detrusor. These factors may lead to aggravation of LUTS (29, 30).

Our results also showed that the PV of the diabetic BPH group was significantly higher than that of the non-diabetic BPH group. Firstly, diabetic patients are often insulin resistant, and insulin resistance can increase the amount of insulin secreted in patients. Insulin is a growth promoting factor, which can trigger cells to proliferate. Both *in vitro* and *in vivo* experiments have confirmed that insulin can promote the proliferation of prostaglandin cells *via* a signal transduction mechanism (31). In addition, increased levels of insulin and insulin-like growth factor receptor-1 increased the risk of presenting with BPH compared to controls, and even could be used to predict prostate size, where larger prostates expressed the highest levels of insulin and IGF-1 (32). Secondly, excessive insulin can reduce the level of free sex hormone binding globulin, allowing more androgen to enter prostate cells, and thus leading to prostatic gland hyperplasia and cell enlargement (33); Thirdly, and increasing number of studies have confirmed that inflammation plays an important role in the development of benign prostatic hyperplasia (34). Systemic inflammation and oxidative stress caused by diabetes can cause prostatic hyperplasia (35).

These results suggest that diabetes may reduce Qmax in BPH patients. The decrease in Qmax indicates that the contractile function of bladder detrusor is weakened or the bladder neck and urethral outlet are narrow or obstructed (28). Diabetes, denoted by high glucose levels, can cause hypertrophy and thus reduce the function of the bladder detrusor (36). Diabetes can cause peripheral nerve dysfunction, which in turn increases bladder sensitivity and uncoordinated detrusor movement (37).

However, this study has some limitations. Due to the lack of randomized controlled trials, the studies in this meta-analysis were only case-control studies. There are huge variations in the course of diabetes and BPH in different studies, resulting in high heterogeneity. In addition, the LUTS of BPH patients may also be affected by many factors, including age, smoking, drinking and lifestyle (38), which may have influenced the results. Therefore, the results of this meta-analysis should be interpreted cautiously.

## Conclusion

This systematic review is the first to evaluate the impact of diabetes mellitus on LUTS in BPH patients. The results of our meta-analysis support the hypothesis that LUTS in BPH patients is increased in patients with diabetes mellitus compared with controls, which suggests that physicians should pay more attention to BPH patients with diabetes mellitus.

## Data Availability Statement

The original contributions presented in the study are included in the article/**Supplementary Material**. Further inquiries can be directed to the corresponding authors.

## Author Contributions

XS designed the study. XS and JX searched databases and collected the data. JX and JH assessed the quality of the study. XS performed the analysis. XS and CX wrote the manuscript. HF and QL modified the manuscript in the revision. All authors contributed to this systematic review and meta-analysis. All authors contributed to the article and approved the submitted version.

## Conflict of Interest

The authors declare that the research was conducted in the absence of any commercial or financial relationships that could be construed as a potential conflict of interest.

## Publisher’s Note

All claims expressed in this article are solely those of the authors and do not necessarily represent those of their affiliated organizations, or those of the publisher, the editors and the reviewers. Any product that may be evaluated in this article, or claim that may be made by its manufacturer, is not guaranteed or endorsed by the publisher.

## Supplementary Material

Supplementary Figure S1 The sensitivity analysis results of International Prostate Symptom Score comparing diabetes group to without diabetes group in benign prostatic hyperplasia patients.

Supplementary Figure S3 The sensitivity analysis results of maximal flow rate comparing diabetes group to without diabetes group in benign prostatic hyperplasia patients.

Supplementary Figure S4 The sensitivity analysis results of prostate-specific antigen value comparing diabetes group to without diabetes group in benign prostatic hyperplasia patients.

Supplementary Figure S5 The funnel plot of International Prostate Symptom Score comparing diabetes group to without diabetes group in benign prostatic hyperplasia patients.

Supplementary Figure S6 The funnel plot of prostate volume comparing diabetes group to without diabetes group in benign prostatic hyperplasia patients.

Supplementary Figure S7 The funnel plot of maximal flow rate comparing diabetes group to without diabetes group in benign prostatic hyperplasia patients.

Supplementary Figure S8 The funnel plot results of prostate-specific antigen value comparing diabetes group to without diabetes group in benign prostatic hyperplasia patients.
