# Supplementary figures and images for: Impact of Diabetes Mellitus on Lower Urinary Tract Symptoms in Benign Prostatic Hyperplasia Patients: A Meta-Analysis
Source: Front Endocrinol (Lausanne). 2022 Feb 1;12:741748. doi: 10.3389/fendo.2021.741748 (PMC8844560; doi:10.3389/fendo.2021.741748)

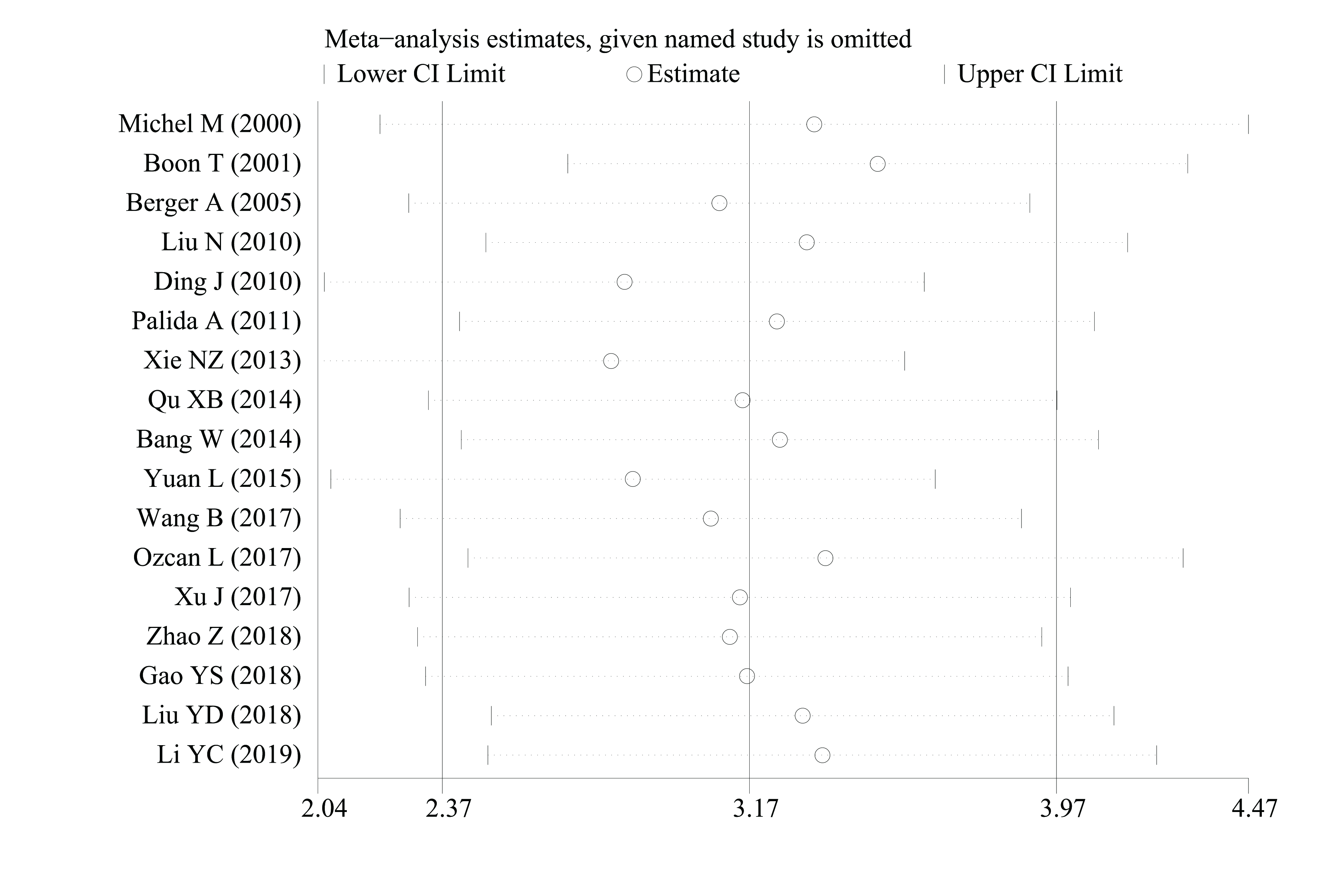

Supplement: Supplementary Figure S1 — The sensitivity analysis results of International Prostate Symptom Score comparing diabetes group to without diabetes group in benign prostatic hyperplasia patients. [file Image_1.tif]

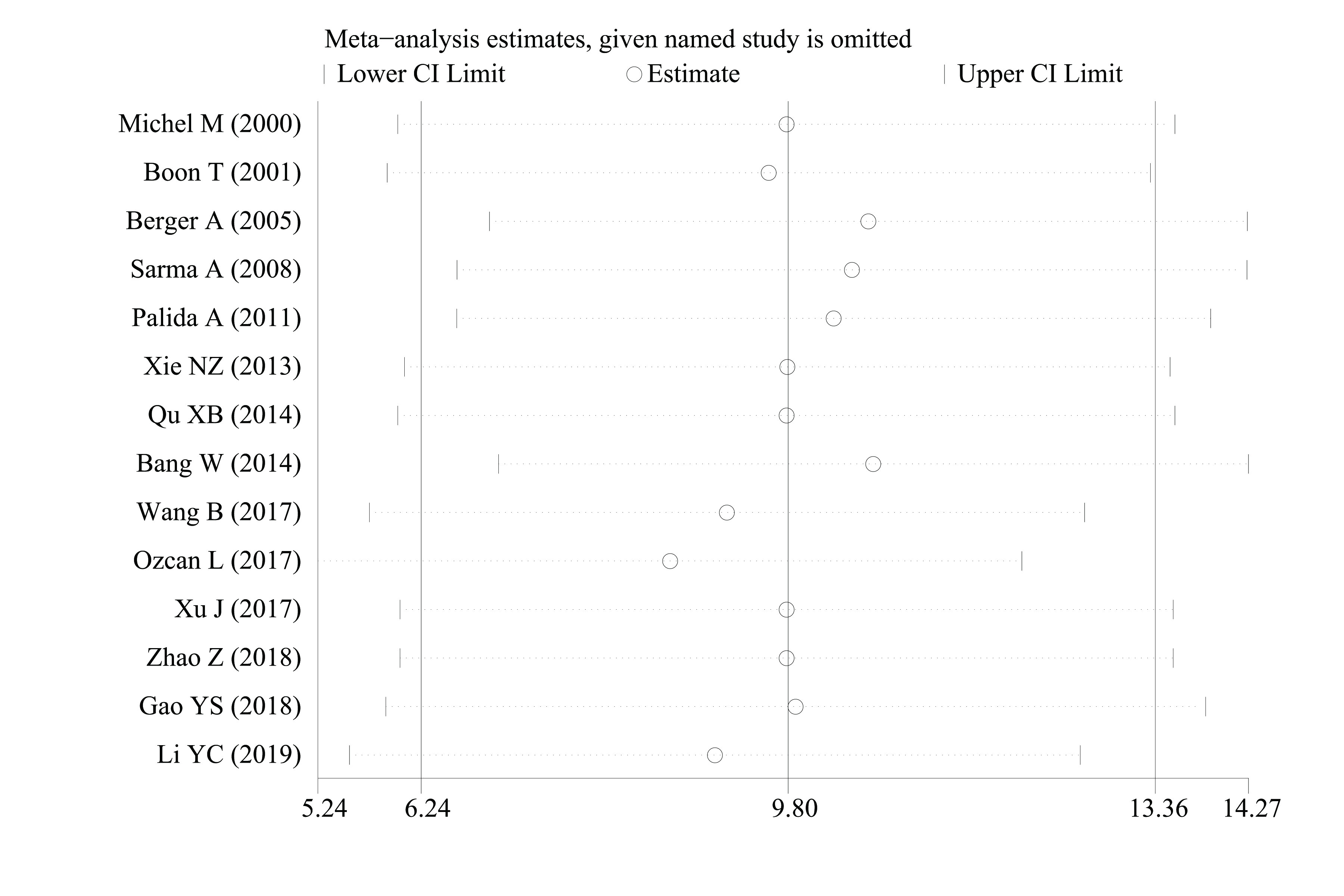

Supplement: Supplementary file 2 [file Image_2.tif]

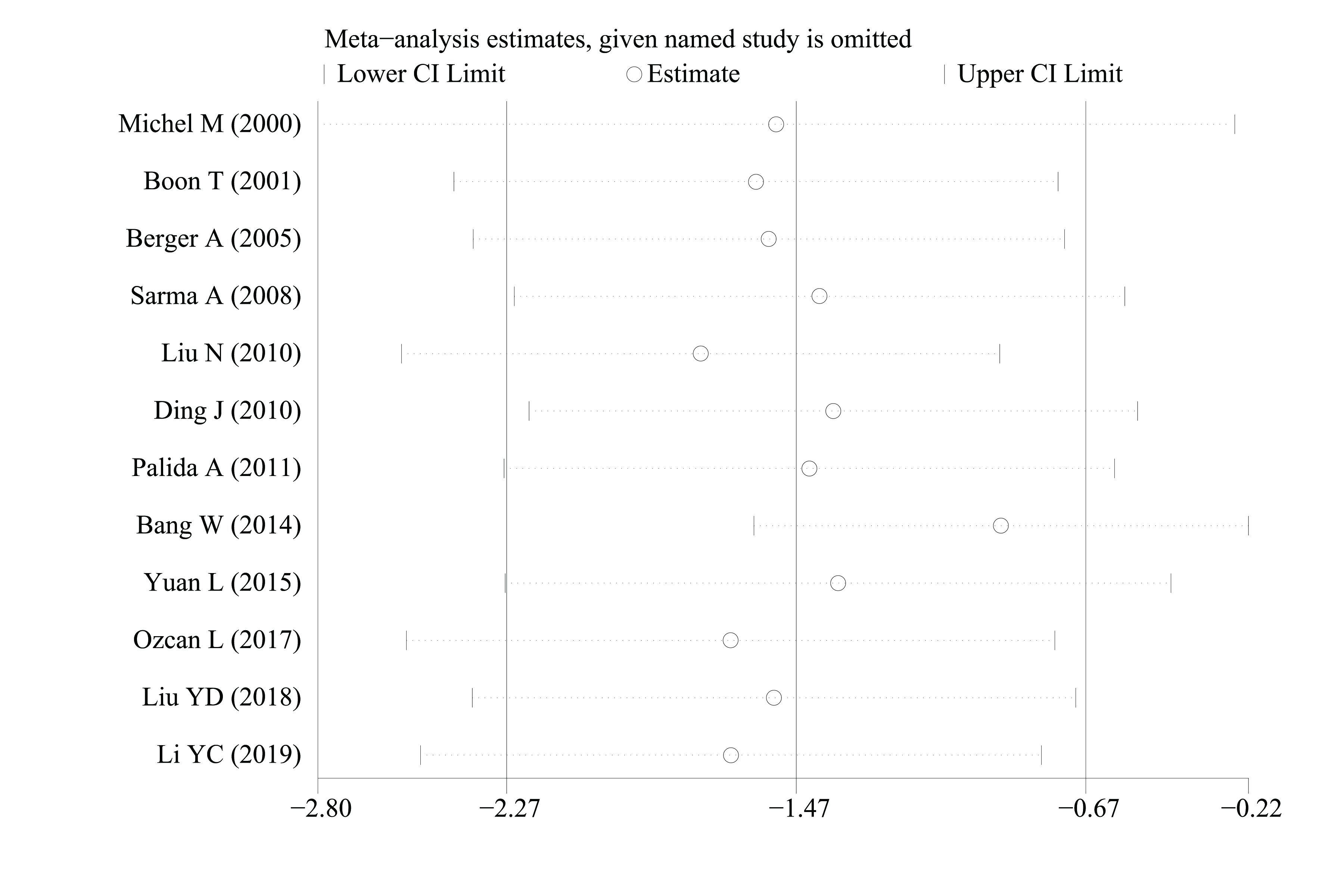

Supplement: Supplementary Figure S3 — The sensitivity analysis results of maximal flow rate comparing diabetes group to without diabetes group in benign prostatic hyperplasia patients. [file Image_3.tif]

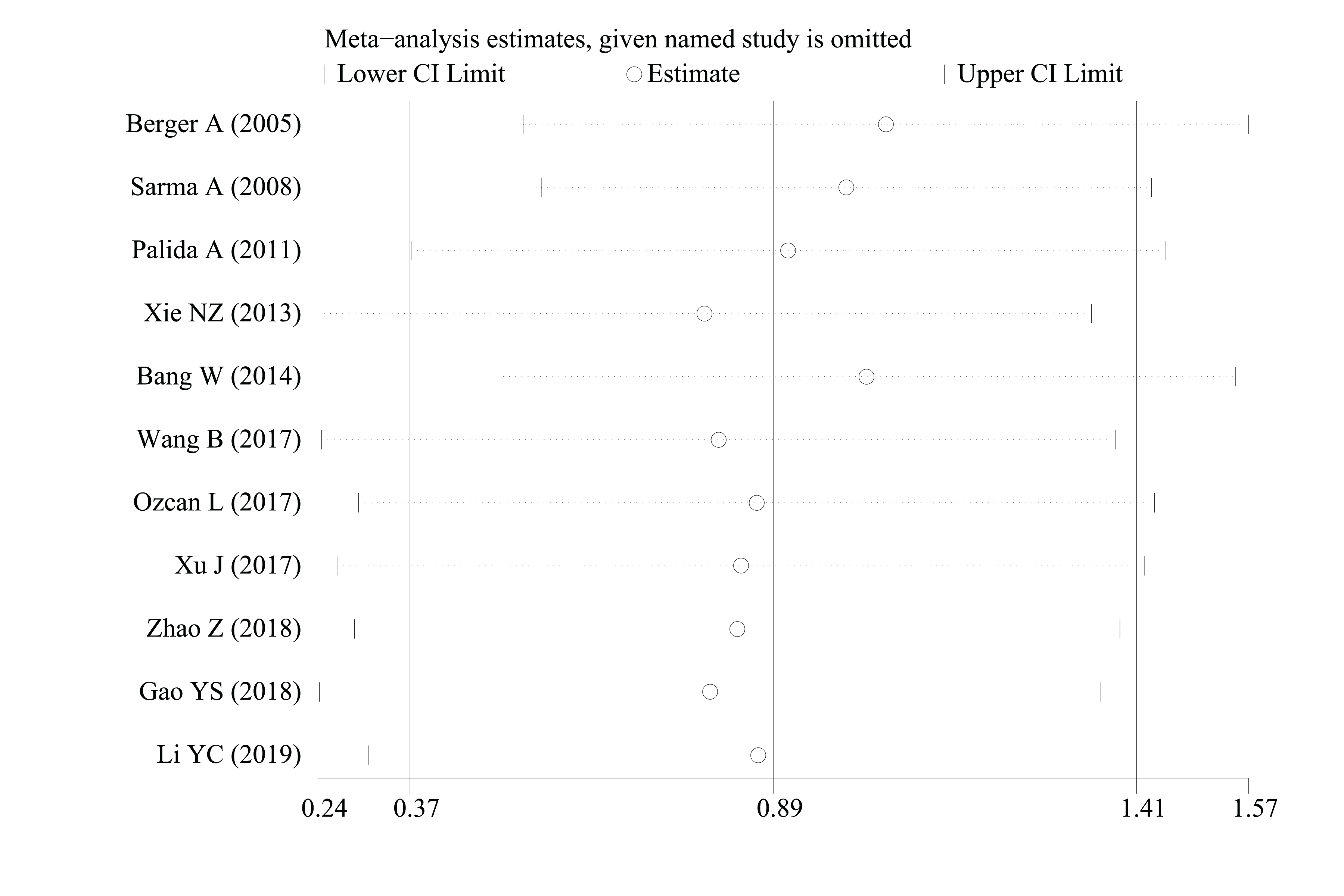

Supplement: Supplementary Figure S4 — The sensitivity analysis results of prostate-specific antigen value comparing diabetes group to without diabetes group in benign prostatic hyperplasia patients. [file Image_4.tif]

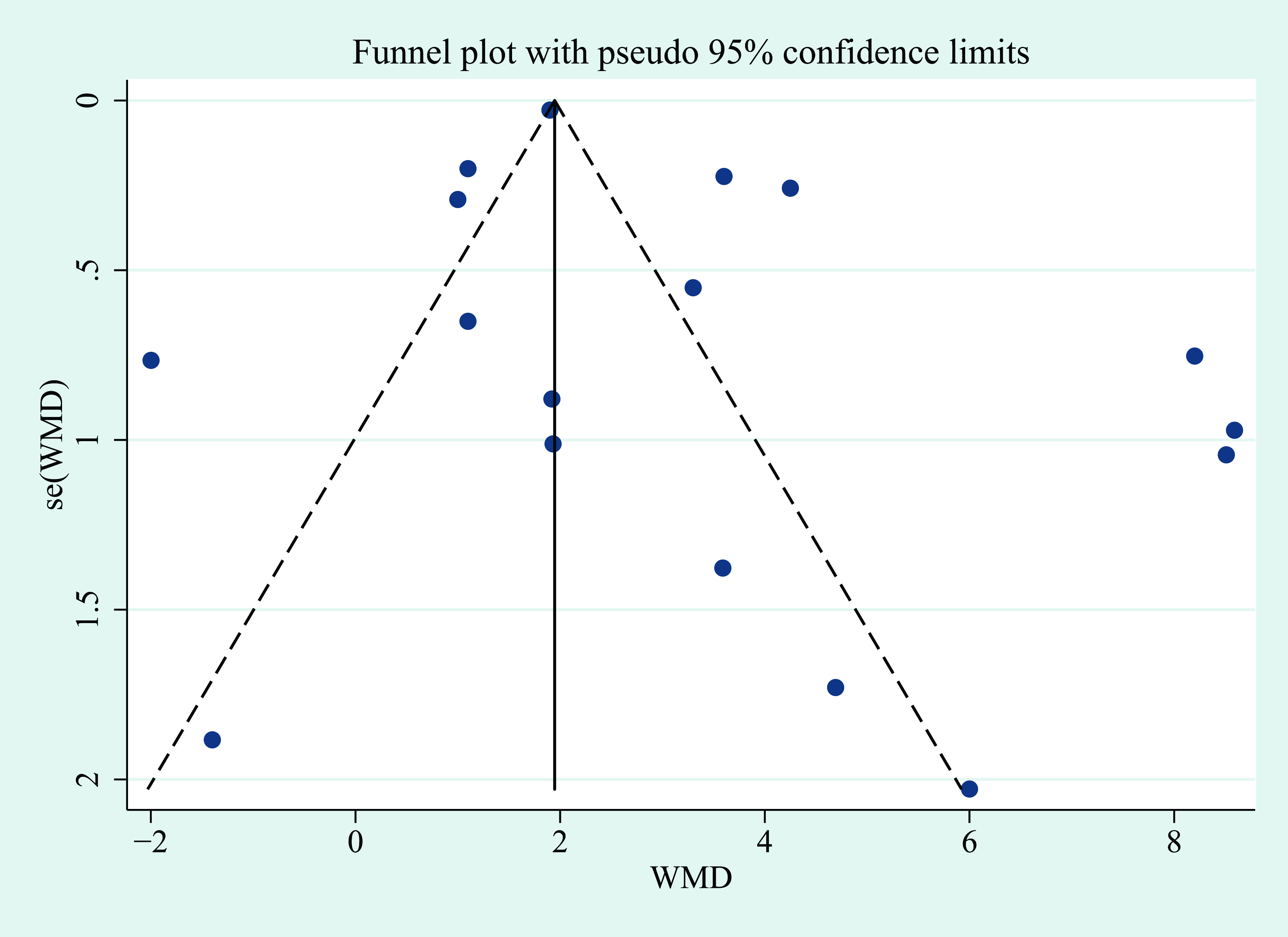

Supplement: Supplementary Figure S5 — The funnel plot of International Prostate Symptom Score comparing diabetes group to without diabetes group in benign prostatic hyperplasia patients. [file Image_5.tif]

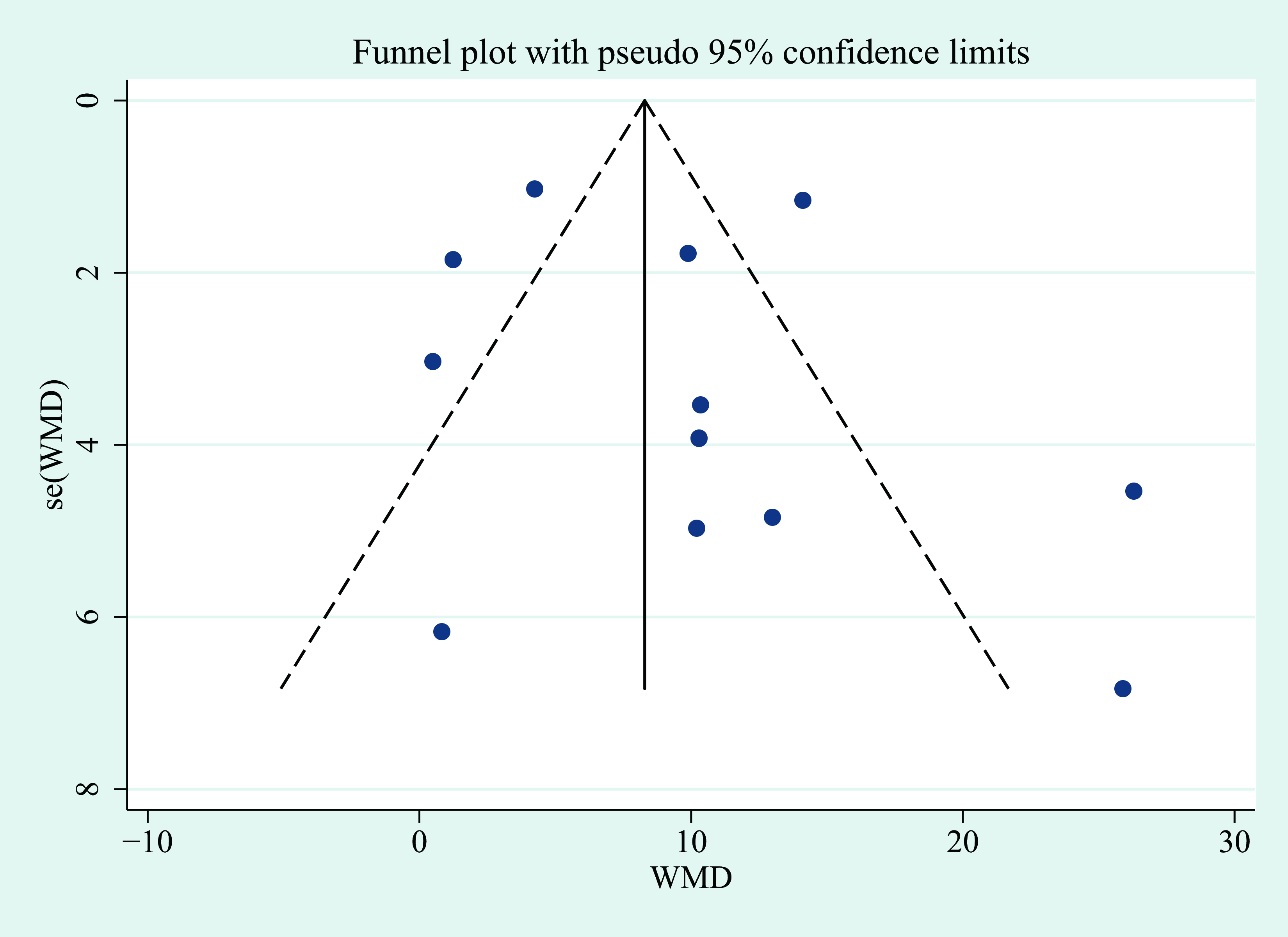

Supplement: Supplementary Figure S6 — The funnel plot of prostate volume comparing diabetes group to without diabetes group in benign prostatic hyperplasia patients. [file Image_6.tif]

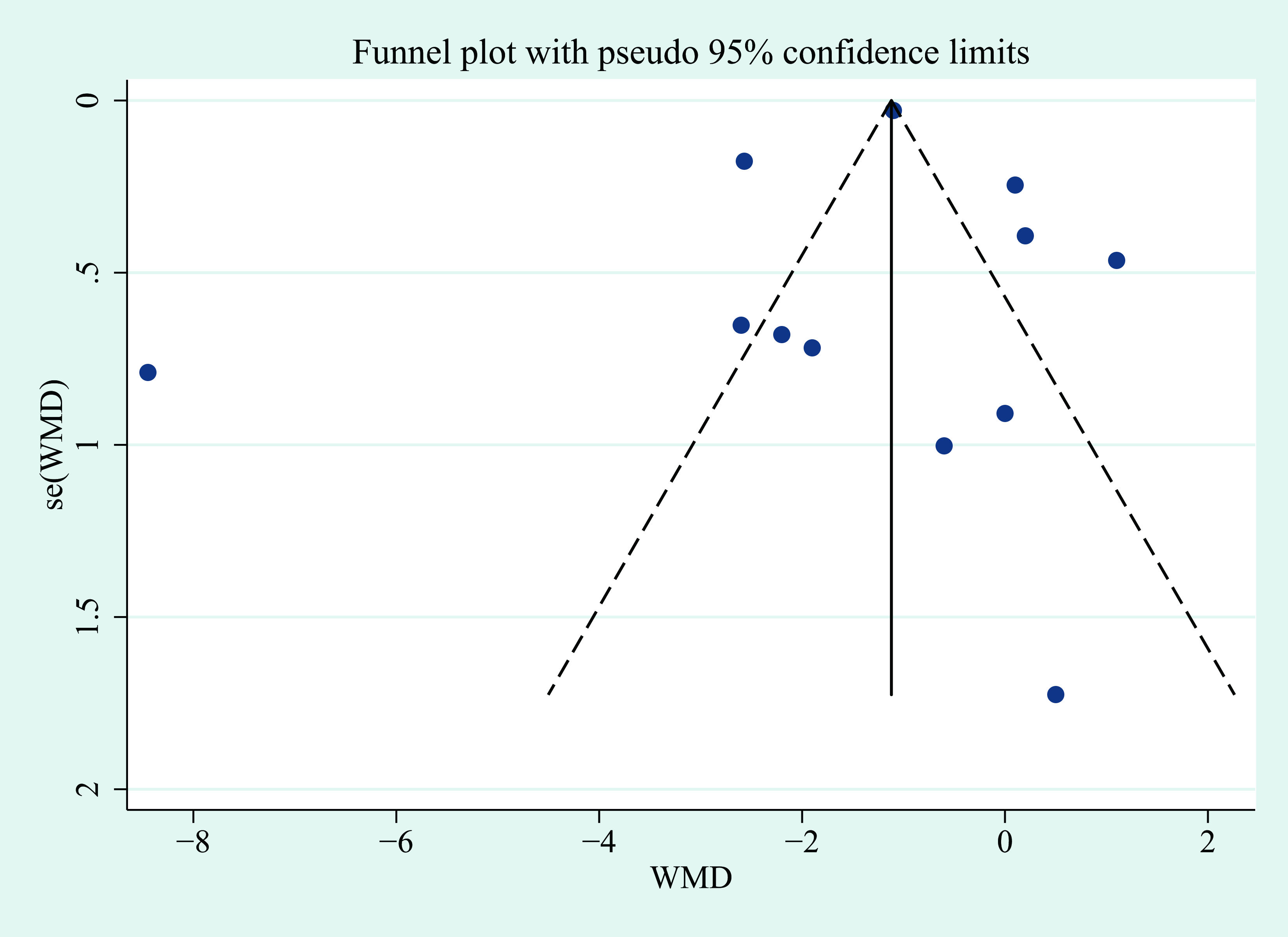

Supplement: Supplementary Figure S7 — The funnel plot of maximal flow rate comparing diabetes group to without diabetes group in benign prostatic hyperplasia patients. [file Image_7.tif]

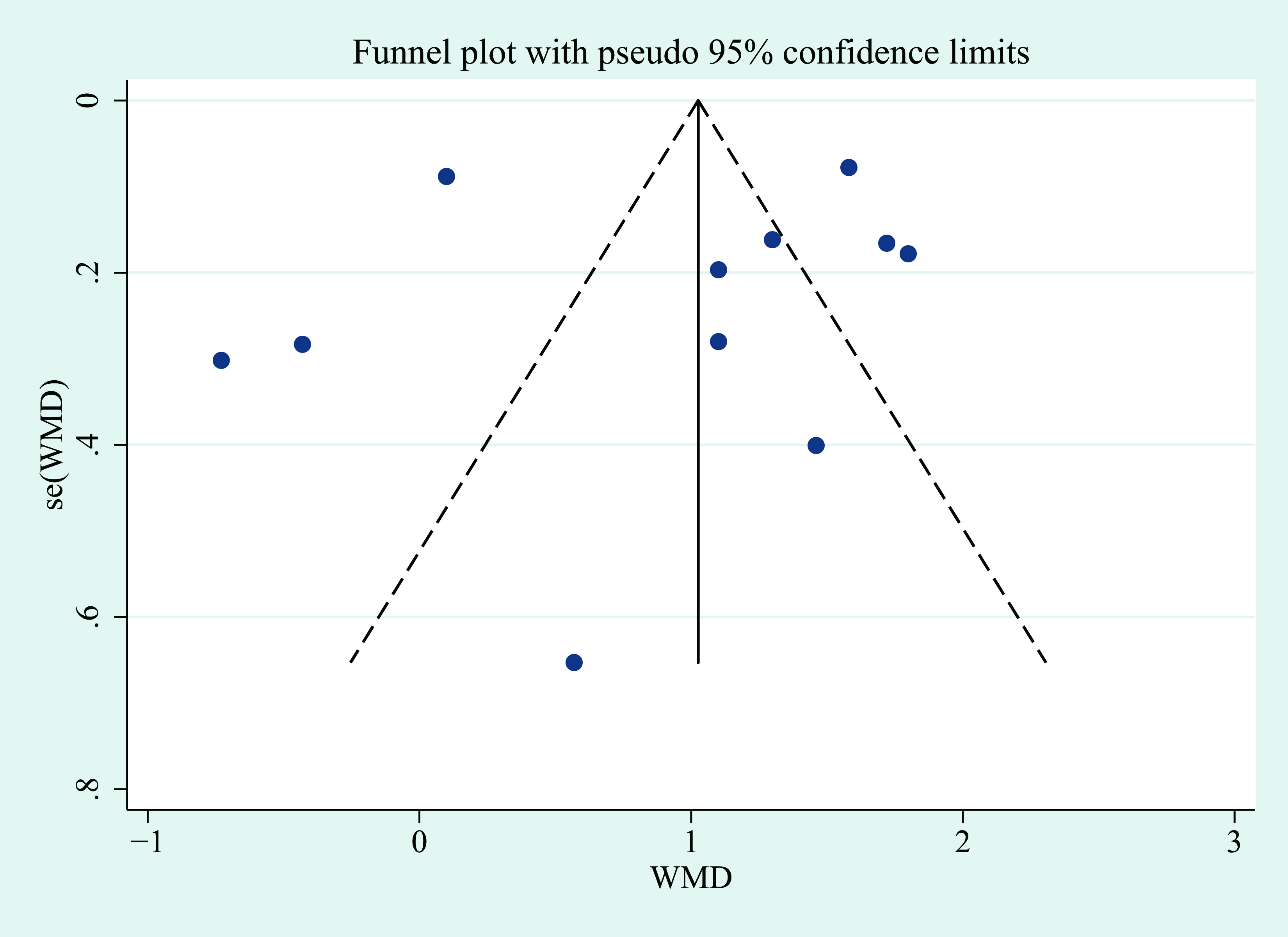

Supplement: Supplementary Figure S8 — The funnel plot results of prostate-specific antigen value comparing diabetes group to without diabetes group in benign prostatic hyperplasia patients. [file Image_8.tif]
