# Supplementary material for: Impact of Diabetes Mellitus on Lower Urinary Tract Symptoms in Benign Prostatic Hyperplasia Patients: A Meta-Analysis
Source: Front Endocrinol (Lausanne). 2022 Feb 1;12:741748. doi: 10.3389/fendo.2021.741748 (PMC8844560; doi:10.3389/fendo.2021.741748)
Supplement: Supplementary file 10 [file Table_2.doc]

**Table S2 The summary of risks based on the NOS.**

| **Study** | **Publication Year** | **NOS** | | |
| --- | --- | --- | --- | --- |
|  |  | **Selection** | **Comparability** | **Exposure** |
| Michel M | 2000 | 3 | 1 | 2 |
| Boon T | 2001 | 3 | 2 | 2 |
| Berger A | 2005 | 3 | 2 | 3 |
| Sarma A | 2008 | 2 | 1 | 3 |
| Liu N | 2010 | 2 | 1 | 2 |
| Ding J | 2010 | 2 | 2 | 2 |
| Palida A | 2011 | 3 | 2 | 2 |
| Xie NZ | 2013 | 2 | 2 | 2 |
| Qu XB | 2014 | 2 | 2 | 3 |
| Bang W | 2014 | 4 | 2 | 3 |
| Yuan L | 2015 | 2 | 1 | 2 |
| Wang B | 2017 | 3 | 2 | 2 |
| Ozcan L | 2017 | 2 | 2 | 3 |
| Xu J | 2017 | 2 | 2 | 3 |
| Zhao Z | 2018 | 2 | 2 | 2 |
| Gao YS | 2018 | 3 | 2 | 2 |
| Liu YD | 2018 | 3 | 1 | 2 |
| Li YC | 2019 | 3 | 2 | 2 |

NOS: Newcastle-Ottawa Scale.
